# Ovariotomy

**Published:** 1889-10

**Authors:** 


### OVARIOTOMY.

In view of some complications connected with an ovariotomy performed in this city July 27th, 1889, by Dr. J. McF. Gaston, with the assistance of Drs. G. G. and C. D. Roy and Dr. J. G. Earnest, a brief report of the operation may prove of interest to the profession.

A white lady fifty-nine years old, had given birth to a son who is thirty years of age, and had been a widow for quite awhile, when there appeared an enlargement in the left side of the abdomen. Within the past two years this gradually increased so as to fill up the abdomen. In view of the fluctuation upon palpation, it was treated for dropsy by so-called specialists, until the distension reached the utmost capacity of the abdominal walls. The case was diagnozed by Dr. G. G. Roy as an ovarian cyst, and supported by Dr. Gaston; yet in view of the fluctuation throughout the entire abdominal region, it was thought prudent to make a small incision at the outset, into the peritonial cavity. Upon cutting into the linea alba, midway between the umbilicus and pubes, a dense attachment of the peritoneal coats precluded a separation with the finger, and in the dissection the sac was entered unintentionally by a small aperture, giving an outlet to a dark gray fluid, characteristic of ovarian cysts. The large trocar was thrust into this opening and the rubber tubing tied to prevent the escape of the fluid, while the work of enlarging the incision and breaking up the attachments was completed with much difficulty.

The peritoneal lining of the abdomen was so firmly adherent to the anterior and lateral surface of the tumor that considerable force was required in the separation, leaving the peritoneal surface raw and oozing blood at every point from the capillaries.

After discharging a portion of the fluid through the trocar the sac was drawn into the incision and laid open with the bistoury, giving a free outlet to the remaining contents, when it was found that a solid mass with numerous gelatinous cysts formed the base of the tumor.

The pedicle was transfixed by a large curved needle carrying a number 14 irondyed silk ligature. This was cut and tied firmly on each side, and then secured around the whole pedicle by carrying the doubled threads in opposite directions and knotting them. The pedicle was divided with scissors ¾ of an inch outside of the ligature and the surface touched with pure carbolic acid as a corrective. Hot carbolized water being poured into the abdominal cavity, and emptied out by turning the patient on her side, sponges were employed to remove the remaining fluid, when it was discovered that blood was flowing from a vessel near the pedicle. By passing a needle with a ligature through the tissues and tying on either side, with an encircling loop around the mass, the hemorrhage was controlled. All blood was removed by sponges, and the abdomen closed without drainage by twenty stitches of interrupted suture throughout the structures.

Iodoform was dusted over the line of the incision extending from the umbilicus to the mons veneris idoform gauze was laid over this, and then a thick compress of absorbent cotton over the entire abdomen was secured by a flannel roller.

The patient rallied well, and after the effects of the A. C. E. passed off, a hypodermic of morphia, gr. $\frac{1}{4}$, with atropia 1-150 was used to secure rest.

Every alternate stitch was removed on Aug. 2nd, and the others on the 5th without any indication of pus even in the tracks of the stitches. But subsequently the cutaneous union yielded, and there was slight suppuration along the entire line, without any communication with the abdominal cavity. All healed kindly in the course of a month, and at the end of two months the patient is entirely free from trouble of any kind.

